# Describing the natural history of clinical, biochemical and radiological outcomes of children with familial partial lipodystrophy type 2 (FPLD2) from the United Kingdom: A retrospective case series

**DOI:** 10.1111/cen.14806

**Published:** 2022-08-17

**Authors:** Zhu Xuan Zhong, Julie Harris, Ellen Wilber, Samantha Gorman, David B. Savage, Stephen O'Rahilly, Anna Stears, Rachel M. Williams

**Affiliations:** ^1^ University of Cambridge Cambridge UK; ^2^ Cambridge University Hospitals NHS Trust Cambridge UK; ^3^ Institute of Metabolic Science University of Cambridge Cambridge UK

**Keywords:** children, familial partial lipodystrophy, insulin resistance, laminopathies, lipid metabolism disorders, lipodystrophy, metabolic diseases

## Abstract

**Context:**

Familial partial lipodystrophy type 2 (FPLD2) results from autosomal dominant mutations in the LMNA gene, causing lack of subcutaneous fat deposition and excess ectopic fat accumulation, leading to metabolic complications and reduced life expectancy. The rarity of the condition means that the natural history of FPLD2 throughout childhood is not well understood. We report outcomes in a cohort of 12 (5M) children with a genetic diagnosis of FPLD2, under the care of the UK National Severe Insulin Resistance Service (NSIRS) which offers multidisciplinary input including dietetic, in addition to screening for comorbidities.

**Objective:**

To describe the natural history of clinical, biochemical and radiological outcomes of children with FPLD2.

**Design:**

A retrospective case note review of children with a genetic diagnosis of FPLD2 who had been seen in the paediatric NSIRS was performed.

**Patients:**

Twelve (5M) individuals diagnosed with FPLD2 via genetic testing before age 18 and who attended the NSIRS clinic were included.

**Measurements:**

Relationships between metabolic variables (HbA1c, triglycerides, fasting insulin, fasting glucose and alanine transaminase [ALT]) across time, from first visit to most recent, were explored using a multivariate model, adjusted for age and gender. The age of development of comorbidities was recorded.

**Results:**

Three patients (all female) developed diabetes between 12 and 19 years and were treated with Metformin. One female has hypertrophic cardiomyopathy and four (1M) patients developed mild hepatic steatosis at a median [range] age of 14(12–15) years. Three (1M) patients reported mental health problems related to lipodystrophy. There was no relationship between biochemical results and age. Patients with diabetes had higher concentrations of ALT than patients who did not have diabetes, adjusted for age, gender and body mass index standard deviation scores.

**Conclusions:**

Despite dietetic input, some patients, more commonly females, developed comorbidities after the age of 10. The absence of relationships between biochemical results and age likely reflects a small cohort size. We propose that, while clinical review and dietetic support are beneficial for children with FPLD2, formal screening for comorbidities before age 10 may not be of benefit. Clinical input from an multidisciplinary team including dietician, psychologist and clinician should be offered after diagnosis.

## 1 BACKGROUND

Lipodystrophy syndromes, which may be congenital or acquired, are a group of disorders where there is a selective deficiency of adipose tissue. They are associated with potentially serious metabolic comorbidities due to ectopic fat accumulation, which includes insulin‐resistant diabetes, hypertriglyceridaemia and hepatic steatosis.^1^ Lipodystrophy is further subclassified as generalized or partial, based on the pattern and extent of fat loss.

Familial partial lipodystrophy 2 (FPLD2), or Dunnigan variety lipodystrophy, is an autosomal dominantly inherited condition that results from pathogenic variants in the LMNA gene,^2^, ^3^, ^4^, ^5^ located on chromosome 1q21.^6^, ^7^ The gene encodes for lamin A and the splice‐variant lamin C, which are intermediate filament proteins that form the nuclear lamina and provide structural stability to the nuclear envelope.^8^, ^9^ Depending on the site of the variant, individuals exhibit different severity of phenotype.^10^ The exact disease mechanisms are unknown, but it is postulated that mutations within or near the lamin DNA binding domain alter interactions of the transcription factors or other DNA binding molecules to cause adipocyte death.^9^ Capanni et al.^11^ found that lamin A precursors specifically accumulated in lipodystrophic cells, and sequesters the adipocyte transcription factor sterol regulatory element binding protein 1 at the nuclear rim, preventing them from activating the peroxisome proliferator‐activate receptor (PPARgamma), thereby impairing preadipocyte differentiation.

The classic physical phenotype described is failure of subcutaneous fat deposition in the distal and truncal regions, with excess accumulation of fat on the neck and face, causing a Cushingoid appearance that is limited to the face.^10^, ^12^ The phenotype manifests around or shortly before puberty, so young children and infants (particularly males) with FPLD2 may be challenging to distinguish from unaffected individuals.^13^, ^14^, ^15^ Other clinical features include prominent musculature, prominent veins and acanthosis nigricans.^14^

By far the most commonly associated metabolic complications of FPLD2 are impaired glucose tolerance, insulin resistance, hypertriglyceridaemia, hypertension and hepatic steatosis.^10^, ^12^, ^16^, ^17^, ^18^ Several studies also report a high prevalence of pancreatitis, especially in females, likely secondary to hypertriglyceridaemia.^4^, ^14^, ^18^ There have also been reports that biochemical parameters such as triglycerides vary with age.^19^ Patients with FPLD2 have an increased risk of premature (under 55 years) coronary artery disease, atherosclerosis,^15^, ^20^ and increased frequency of myocardial infarction earlier in life,^21^ likely a consequence of insulin resistance and dyslipidaemia. Other reported complications include muscular hypertrophy,^22^ nonspecific degenerative joints and tendon abnormalities,^23^ raised inflammatory cytokines,^16^ painful musculoskeletal conditions and mood disorders requiring medication.^18^ Additionally, affected women have also been reported to have a higher incidence of polycystic ovarian syndrome, and may have significant and distressing hirsutism.^15^, ^18^, ^24^ It should be noted that men seem to present less frequently clinically,^14^, ^25^ despite an autosomal dominant inheritance pattern.

International consensus recommends that affected individuals should receive annual screening for diabetes mellitus, hypertension and hypertriglyceridaemia; liver ultrasound, echocardiogram and measurement of gonadal steroids should be performed as clinically indicated.^12^

Currently, the first line and primary mode of management is a low‐fat diet,^12^, ^13^ though leptin replacement therapy has been shown to be useful in the management of metabolic complications where conventional therapy for diabetes and dyslipidaemia have been unsuccessful, helping to reduce HbA1C and triglycerides levels.^26^, ^27^, ^28^, ^29^, ^30^ However, recombinant leptin may not be as effective in patients with FPLD compared to patients with generalized lipodystrophy, given that the former have significantly higher concentrations of leptin.^26^, ^30^ Leptin treatment has recently been approved by the European Medicines Agency (EMA), the Food and Drug Administration (FDA) and the National Institute for Health and Care Excellence (NICE) for the treatment of patients with FPLD2 in whom conventional therapies for diabetes and hypertriglyceridaemia have failed.

The National Severe Insulin Resistance Service (SIRS) at Addenbrooke's Hospital was set up in 2012 and serves as the national centre for following up all patients with lipodystrophy in the United Kingdom. Children with FPLD2 are offered a package of care that includes ideally: annual review with a specialist paediatric endocrinologist, annual screening for comorbidities and specialist dietetic input to ensure a low‐fat diet.

Dietary advice and counseling is provided to children and their families to help minimize the risk of metabolic complications such as diabetes, dyslipidaemia and fatty liver. Adhering to a low fat, healthy diet and avoiding excess dietary energy, while allowing normal growth, is the mainstay of treatment.^31^

The level of dietary restriction depends on the specific diagnosis and biochemistry and should consider the patients' cultural, social and financial needs.

There is a paucity of published data on the effects of dietary manipulation in lipodystrophy, particularly within paediatrics. Therefore, current suggestions are based on limited sources and anecdotal evidence. Much of the advice given comes from an evidence base for conditions such as type 2 diabetes, obesity, cardiovascular disease and hypertriglyceridemia.

Historically, diagnosis was made using targeted genetic testing when there was clinical suspicion. However, with the advent of more widely available genetic testing, there is an opportunity to screen the children of indexes in early life before onset of symptoms, allowing close monitoring with the aim of improving health outcomes through early intervention.

## 2 OBJECTIVES

To describe clinical, biochemical and radiological outcomes in children with FPLD2, in addition to comorbidities, additional pathologies and therapies and explore their relationships with age and gender.

## 3 LITERATURE SEARCH

A search on PubMed using the terms (paediatrics OR child OR children OR infant OR paediatric OR neonatal OR neonates) AND (‘familial partial lipodystrophy’ OR Dunnigan OR FPLD) with a filter for ‘humans’ returned 142 results. Of the relevant literature, while some studies have been done to examine the clinical and biochemical parameters of children with FPLD2 at a single point in time, no studies have examined how those parameters may vary with time in children with FPLD2. Hence, this paper serves as one of the first to describe the natural history of FPLD2 progression in children.

## 4 METHODOLOGY

Following institutional approval, data on patients with FPLD2 attending the pediatrics division of the National Severe Insulin Resistance Service (SIRS) at Addenbrooke's Hospital (Table 1) were obtained from the electronic patient database and anonymized at collection. All patients had an affected parent, and the diagnosis was made using genetic testing. Thereafter, the team has aimed to offer annual follow‐up appointments, with screening for comorbidities using blood tests and ultrasound, as well as offering specialist dietetic input to support adherence to a low‐fat diet.

**Table 1:** Ages of children with FPLD2 attending the paediatrics division of the National SIRS at Addenbrooke's Hospital, Cambridge, UK

	Gender	Age at 1st visit	Age at most recent follow‐up	No. of follow‐ups	Years of follow‐up
1	M	11.6	13.6	3	2
2	F	6.2	10.5	3	4
3	F	14.7	21.2	4	7
4	M	9.3	16.4	6	7
5	F	11.2	18.6	8	7
6	M	9.1	16.1	7	7
7	F	4.7	11.7	7	7
8	F	14.4	18.2	3	4
9	F	10.2	12.6	5	2
10	M	16.1	17.3	2	1
11	F	8.0	9.1	2	1
12	M	12.3	18.7	8	6

Abbreviation: FPLD2, familial partial lipodystrophy type 2.

Data collected includes measures of growth (height, weight and body mass index [BMI] that were converted into standard deviation [SD] scores using the LMS method^32^), medical history, blood test results (liver function test, fasting lipids, blood glucose, HbA1C, fasting insulin) and imaging results (echocardiogram scans, abdominal ultrasound scans).

**Figure 1:**
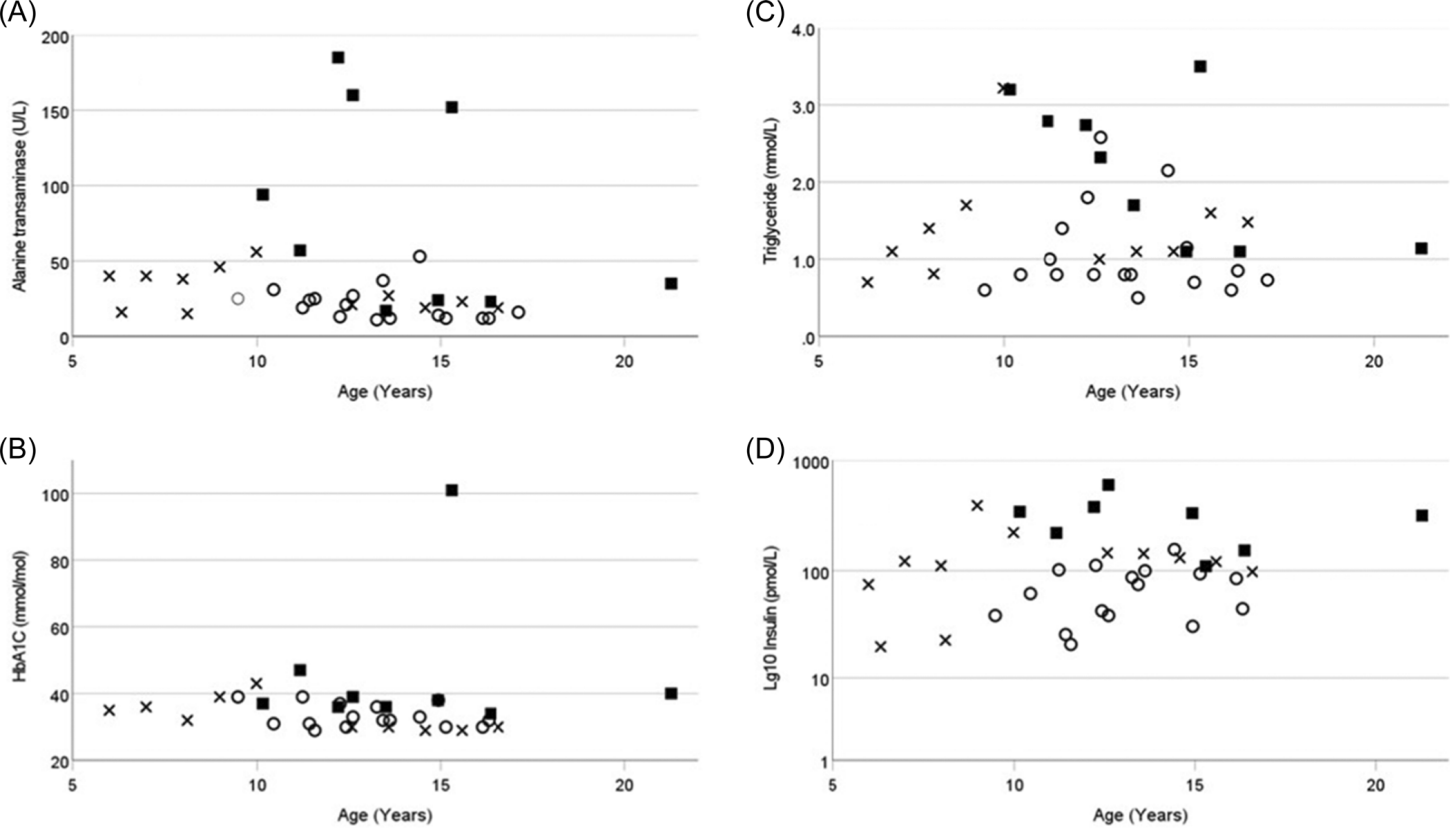
Metabolic parameters for all patients plotted against decimal age in years. Males—open circles, females—open triangles, females with diabetes—filled squares, Panel (A) alanine transaminase, (B) HbA1c, (C) triglycerides, (D) Log(10) insulin.

Metabolic parameters were represented using scatter plots against decimal age (Figure 1).

Statistical analysis of the fully anonymized data set was performed using SPSS. Insulin and triglyceride concentrations were transformed (Log10) to permit parametric testing. Relationships between dependent variables (HbA1c, triglycerides, fasting insulin, fasting glucose and alanine transaminase [ALT]) were explored using a multivariate model incorporating subject ID and gender as fixed factors, with decimal age as a covariate. Relationships between fasting insulin and triglyceride levels were explored using bivariate correlation. A *p* < .05 (two‐tailed) was considered significant.

Differences in dependent variables between patients with and without diabetes were explored using a multivariate model incorporating gender and diabetes diagnosis as fixed factors, BMI SD score and decimal age as covariates. A *p* < .01 (two‐tailed) was considered significant for this subgroup analysis.

## 5 RESULTS

Data are expressed as median [range], median (interquartile range) or mean ± SD.

Data from 12 patients (seven females) are presented. Age at most recent follow‐up was 16.3 [9.1–21.3] years. Demographic data are summarized in Table 1. The duration of follow‐up was 4.5[2–8] years.

**Figure 2:**
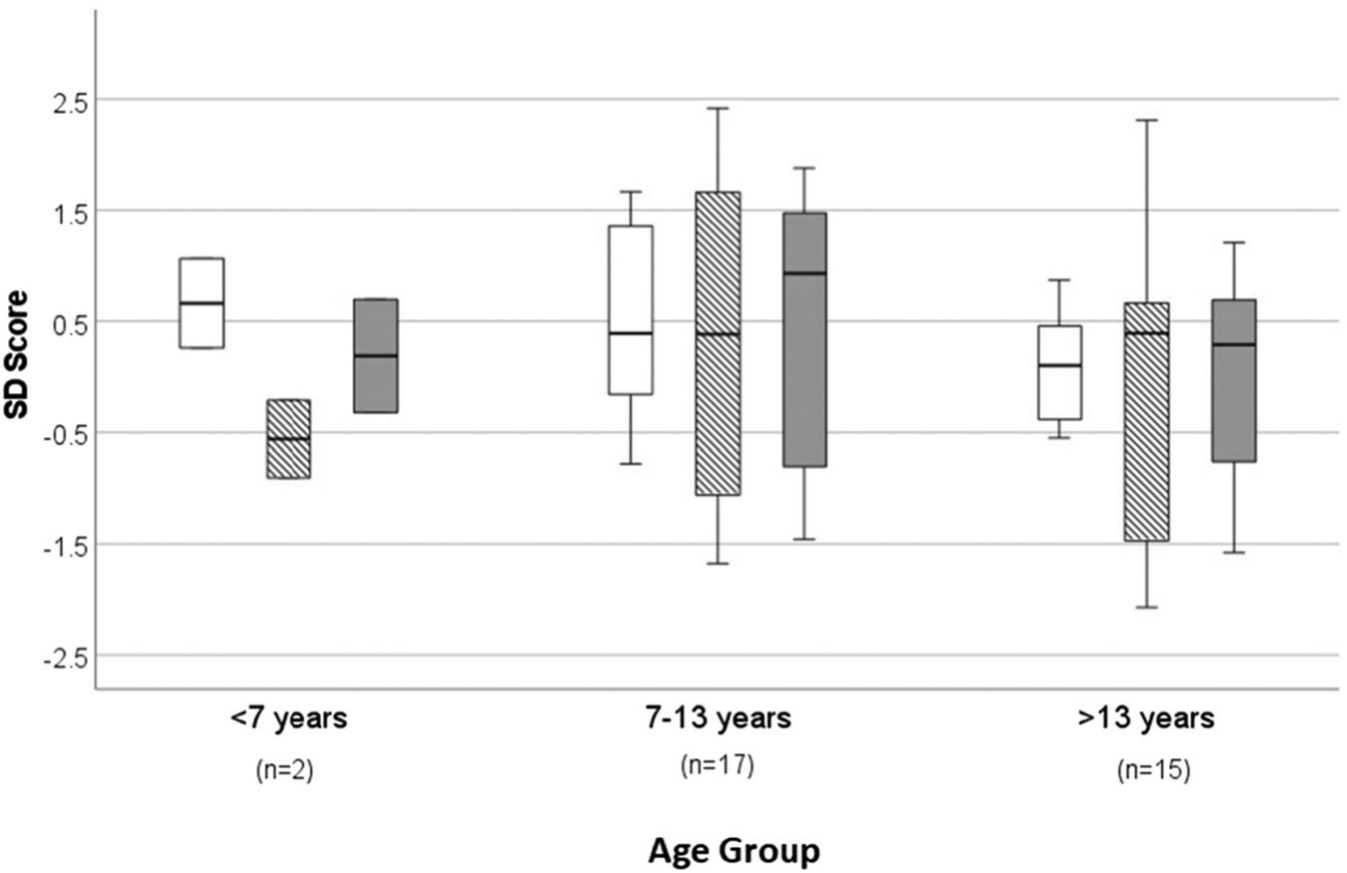
Auxological parameters (BMI SDS [unshaded bars], height SDS [hatched bars] and weight SDS [grey bars]) for all patients by age group represented as box plot—median and interquartile range. BMI, body mass index; SDS, standard deviation scores.

Auxological parameters (BMI, height and weight) SD scores by age group are shown in Figure 2.

All subjects were confirmed to have the same LMNA variant (NM_170707.3, c.1444C>T, p.(Arg482Trp).

### 5.1 Comorbidities

None of our cohort developed any comorbidities at an age younger than 10 years.

#### 5.1.1 Diabetes

Three females were diagnosed with secondary diabetes between the ages of 12 and 21 years and all three were treated with metformin.

#### 5.1.2 Hepatic steatosis

Four patients (three females) developed mild hepatic steatosis between the ages of 10 and 15, diagnosed on routine ultrasound scans of the liver.

#### 5.1.3 Mental health

Three patients (two females) experienced mental health issues, which has been previously reported in other patients.^18^

#### 5.1.4 Other complications

One patient with diabetes also has Emery–Dreifuss muscular dystrophy and hypertrophic cardiomyopathy, which required cardiac surgeries to reduce symptoms. All other patients had normal echocardiography scans thus far.

One patient with diabetes has acanthosis nigricans.

Anecdotally, the physical phenotype was recognizable on clinical examination (albeit with the knowledge of a genetic diagnosis) in all the females at first visit.

Clinical and biochemical data from the most recent appointment are summarized in Table 2.

**Table 2:** Most recent clinical and biochemical data

	Whole cohort	Males (*n* = 5)	Females (no diabetes) (*n* = 4)	Females (diabetes) (*n* = 3)	Reference ranges
Age (median [range])	14.7 [6.3–21.3]	11.6 [12.6–16.3]	9.1 [6.3–16.6]	15.3 [12.2–21.3]	
Height SDS	0.39 ± 1.57	0.31 ± 1.62	0.00 ± 1.94	1.33 ± 0.78	
Weight SDS	0.37 ± 1.16	0.02 ± 1.05	0.29 ± 1.41	1.33 ± 0.78	
BMI SDS	0.47 ± 0.82	−0.12 ± 0.54*	0.57 ± 0.77	1.27 ± 0.65	
Systolic BP SDS	−0.13 ± 0.95	−0.78 ± 1.28	0.09 ± 0.82	0.24 ± 0.61	
Diastolic BP SDS	1.09 ± 0.89	1.00 ± 1.66	1.17 ± 0.25	0.78 ± 0.39	
Alanine transaminase (ALT) (U/L)	50.0 ± 57.7	23.6 ± 17.6	26.5 ± 19.7	124.0 ± 78.8**	7–40
Triglyceride (mmol/L)	1.75 ± 1.03	1.47 ± 0.86	1.55 ± 1.16	2.46 ± 1.21	0.30–1.80
Total cholesterol	4.6 ± 1.1	3.7 ± 1.2	4.9 ± 0.6	4.7 ± 1.4	
HDL cholesterol (mmol/L)	1.08 ± 0.36	1.09 ± 0.39	1.27 ± 0.36	0.79 ± 0.17	M: >0.75
					F: >0.91
LDL cholesterol (mmol/L)	2.76 ± 0.72	2.60 ± 0.82	2.95 ± 0.65	2.79 ± 0.88	<2.59
Leptin (u/L)	6.6 ± 4.7	3.2 ± 2.3*	7.3 ± 5.0	11.9 ± 2.8	
HbA1C (mmol/mol)	41.8 ± 21.2	33.2 ± 2.9	35.0 ± 7.0	59.0 ± 36.4	<42
Adiponectin (μg/ml)	7.7 ± 3.6	7.6 ± 3.4	8.9 ± 4.0	5.0 ± 2.8	
Insulin (pmol/L) (median [interquartile range])	98 (37–173)	62 (39–85)	60.5 (22–129)	317 (213.5–348)	0–80

*Note*: The reference ranges for HDL and LDL cholesterol are based on the ATP III guidelines.

Abbreviations: ATP III, adult treatment panel III; BMI, body mass index; BP, binding protein; HDL, high density lipoprotein; LDL, low density lipoprotein; SDS, standard deviation scores.

* *p* < .01 versus patients without diabetes, adjusted for gender, decimal age and BMI SD scores.

** *p* < .05 versus all females, adjusted for decimal age.

We found no relationship between age and worsening metabolic parameters for either gender (Figure 1).

Leptin concentrations and BMI SD scores were higher in females than males (*p* < .05), consistent with previous reports^27^, ^33^ (Table 2).

ALT concentrations were higher in patients with diabetes (*p* < .01) than those without, adjusted for gender, BMI SDS and age (*p* < .01) (Table 2).

There was no relationship between fasting triglyceride concentrations and fasting insulin concentrations.

## 6 DISCUSSION

We report clinical and biochemical outcomes in a small cohort of children with a genetic diagnosis of FPLD2 in whom the genetic diagnosis was made at a relatively young age. No comorbidities were detected younger than the age of 10 years, but, thereafter we observed the development of complications including nonalcoholic steatohepatitis and diabetes mellitus.^10^, ^14^ In addition to the input of an multidisciplinary team (MDT), which included dietetic input, these children are likely to have grown up in a household with an existing awareness and appreciation of the dietary management of FPLD2. Dietary management to mitigate visceral fat storage with resultant insulin resistance is the most effective intervention in FPLD2^1^, ^12^, ^13^ and it is perhaps disappointing that, despite awareness and professional dietetic advice, we still observed comorbidities in a number of our patients.

A previous study of children with FPLD2 up to age 18 years reported that the metabolic variables for males were not significantly different from members of a healthy control population, while in females, fasting triglyceride concentrations were raised in both the 7–12 and 13–18 years age groups.^25^ Similarly, in our cohort, females appear to be more severely affected, in that all of the patients who developed diabetes were female.^4^, ^25^ The higher leptin concentrations in females compared is also consistent with previous reports.^33^

Increased insulin resistance is correlated with increased hypertriglyceridaemia^15^ and lower serum adiponectin levels.^33^ However, we found no similar relationships between triglyceride concentrations and insulin levels in our cohort. We did observe a gender difference in BMI SD scores which is consistent with the observed gender difference in leptin concentrations.

In a subgroup analysis, ALT concentrations in the three patients with a diagnosis of diabetes were higher than those without diabetes, independent of gender, BMI SD score and decimal age. All patients with a diagnosis of diabetes were treated with metformin. While hepatitis is an uncommon side effect of metformin treatment, the observed elevated ALT levels in patients with diabetes may reflect a population with a more severe phenotype, who developed diabetes and were then treated with metformin. At least one study has reported reductions in ALT concentrations in patients with type 2 diabetes treated with metformin.^34^

All patients in this cohort had the same LMNA variant p.(Arg482Trp). but there was marked variation in the severity of the phenotype. This may reflect the impact of other parameters, for example, apolipoprotein E (apoE) genotype can affect the severity of dyslipidaemia, with the apoE 2/3 genotype being associated with an earlier onset and more pronounced form of dyslipidaemia than those with the apoE 3/3 genotype.^19^ Further work to consider the impact of other variables on phenotypic severity in FPLD2, such as ApoE genotype, would be of interest.

The lack of observed differences in metabolic outcomes may reflect the small sample size. Given the rarity of the condition, an international collaboration to collect outcome data would be helpful to meaningfully inform practice.

FPLD2 belongs to a family of laminopathies, characterized by mutations in the LMNA gene. Other laminopathies include the autosomal dominant Emery–Dreifuss muscular dystrophy, which was seen in one of our patients, LMNA‐dependent dilated cardiomyopathy, and Hutchinson–Guilford progeria syndrome, a severe premature ageing syndrome.^35^, ^36^ Manifestations may be isolated or multisystem, and cardiomyopathy often occurs in those with muscular dystrophy.^35^, ^37^ Cardiomyopathy has not been reported to frequently occur in FPLD2 patients, as the FPLD mutation affects the C‐terminal domain of the LMNA gene, whereas cardiomyopathy mutations affect the rod domain.^2^, ^38^

A number of patients also reported significant psychological distress related especially to their physical appearance and perceived body image, which has also been reported in a previous study.^19^ Data pertaining to the timing of the onset of puberty, hirsutism and other features of polycystic ovarian syndrome were not collected.

Although our study suggests that screening for metabolic complications below the age of 10 years may not be necessary, the severity of the metabolic complications, with resultant impact on quality of life and life expectancy in individuals with FPLD2, means that there is a moral argument for genetic screening of the children of FPLD2 patients to permit early dietary intervention and identification of complications. However, given the absence of complications in early and mid‐childhood, it might be that younger children with FPLD2 may not require screening with annual blood tests and ultrasounds, as long as they are having regular input from a quorate MDT with the necessary dietetic expertize and support. Collation of data from a larger cohort of patients would be required to confirm this.

## 7 CONCLUSION

In our small cohort, despite dietic input, patients were found to develop comorbidities after the age of 10 years, and were more common in females. We suggest that formal screening of comorbidities before age 10 may not be necessary, but clinical input from a multidisciplinary team that includes dietician, psychologist and pediatrician should be offered from diagnosis.

## CONFLICT OF INTEREST

The authors declare no conflict of interest.

## Data Availability

Institutional approval was given for the analysis and reporting of anonymized data collected as part of routine clinical care but we do not have consent from patients to make the data set publicly available.

## References

[1] Garg A . Chapter 23—lipodystrophies. In: Weiss RE , Refetoff S , eds. Genetic Diagnosis of Endocrine Disorders. 2nd ed. Academic Press; 2016:325‐339. 10.1016/B978-0-12-800892-8.00023-3

[2] Speckman RA , Garg A , Du F , et al. Mutational and haplotype analyses of families with familial partial lipodystrophy (dunnigan variety) reveal recurrent missense mutations in the globular C‐terminal domain of lamin A/C. Am J Hum Genet. 2000;66(4):1192‐1198. 10.1086/302836 10739751 PMC1288186

[3] Cao H , Hegele RA . Nuclear lamin A/C R482Q mutation in Canadian kindreds with Dunnigan‐type familial partial lipodystrophy. Hum Mol Genet. 2000;9(1):109‐112. 10.1093/hmg/9.1.109 10587585

[4] Vigouroux C , Magré J , Vantyghem MC , et al. Lamin A/C gene: sex‐determined expression of mutations in Dunnigan‐type familial partial lipodystrophy and absence of coding mutations in congenital and acquired generalized lipoatrophy. Diabetes. 2000;49:1958‐1962. 10.2337/diabetes.49.11.1958 11078466

[5] Shackleton S , Lloyd DJ , Jackson SN , et al. LMNA, encoding lamin A/C, is mutated in partial lipodystrophy. Nat Genet. 2000;24(2):153‐156. 10.1038/72807 10655060

[6] Peters JM , Barnes R , Bennett L , Gitomer WM , Bowcock AM , Garg A . Localization of the gene for familial partial lipodystrophy (Dunnigan variety) to chromosome 1q21‐22. Nat Genet. 1998;18(3):292‐298. 10.1038/ng0398-292 9500556

[7] Wydner KL , McNeil JA , Lin F , Worman HJ , Lawrence JB . Chromosomal assignment of human nuclear envelope protein genes LMNA, LMNB1, and LBR by fluorescence in situ hybridization. Genomics. 1996;32(3):474‐478. 10.1006/geno.1996.0146 8838815

[8] Fisher DZ , Chaudhary N , Blobel G . cDNA sequencing of nuclear lamins A and C reveals primary and secondary structural homology to intermediate filament proteins. Proc Natl Acad Sci. 1986;83(17):6450‐6454. 10.1073/pnas.83.17.6450 3462705 PMC386521

[9] Hegele RA , Joy TR , Al‐attar SA , Rutt BK . Lipodystrophies: windows on adipose biology and metabolism. J Lipid Res. 2007;48(7):1433‐1444. 10.1194/jlr.R700004-JLR200 17374881

[10] Akinci B , Sahinoz M , Oral E . 2018. *Lipodystrophy syndromes: presentation and treatment*. Endotext [Internet], NCBI Bookshelf.

[11] Capanni C , Mattioli E , Columbaro M , et al. Altered pre‐lamin A processing is a common mechanism leading to lipodystrophy. Hum Mol Genet. 2005;14(11):1489‐1502. 10.1093/hmg/ddi158 15843404

[12] Brown RJ , Araujo‐Vilar D , Cheung PT , et al. The diagnosis and management of lipodystrophy syndromes: a multi‐society practice guideline. J Clin Endocrinol Metab. 2016;101:4500‐4511. 10.1210/jc.2016-2466 27710244 PMC5155679

[13] Dunnigan MG , Cochrane MA , Kelly A , Scott JW . Familial lipoatrophic diabetes with dominant transmission. A new syndrome. Q J Med. 1974;43(169):33‐48. 4362786

[14] Gupta N , Asi N , Farah W , et al. Clinical features and management of non‐HIV‐related lipodystrophy in children: a systematic review. J Clin Endocrinol Metab. 2017;102:363‐374. 10.1210/jc.2016-2271 27967300 PMC6283440

[15] Vantyghem MC , Pigny P , Maurage CA , et al. Patients with familial partial lipodystrophy of the Dunnigan type due to a LMNA R482W mutation show muscular and cardiac abnormalities. J Clin Endocrinol Metab. 2004;89:5337‐5346. 10.1210/jc.2003-031658 15531479

[16] Resende ATP , Martins CS , Bueno AC , Moreira AC , Foss‐Freitas MC , de Castro M . Phenotypic diversity and glucocorticoid sensitivity in patients with familial partial lipodystrophy type 2. Clin Endocrinol. 2019;91:94‐103. 10.1111/cen.13984 30954027

[17] Lüdtke A , Genschel J , Brabant G , et al. Hepatic steatosis in Dunnigan‐type familial partial lipodystrophy. Am J Gastroenterol. 2005;100:2218‐2224. 10.1111/j.1572-0241.2005.00234.x 16181372

[18] Ajluni N , Meral R , Neidert AH , et al. Spectrum of disease associated with partial lipodystrophy: lessons from a trial cohort. Clin Endocrinol. 2017;86:698‐707. 10.1111/cen.13311 PMC5395301 28199729

[19] Schmidt HH , Genschel J , Baier P , et al. Dyslipemia in familial partial lipodystrophy caused by an R482W mutation in the LMNA gene. J Clin Endocrinol Metab. 2001;86:2289‐2295. 10.1210/jcem.86.5.7500 11344241

[20] Hegele RA . Premature atherosclerosis associated with monogenic insulin resistance. Circulation. 2001;103:2225‐2229. 10.1161/01.CIR.103.18.2225 11342468

[21] Akinci B , Onay H , Demir T , et al. Clinical presentations, metabolic abnormalities and end‐organ complications in patients with familial partial lipodystrophy. Metabolism. 2017;72:109‐119. 10.1016/j.metabol.2017.04.010 28641778

[22] Akinci G , Topaloglu H , Demir T , et al. Clinical spectra of neuromuscular manifestations in patients with lipodystrophy: a multicenter study. Neuromuscul Disord. 2017;27:923‐930. 10.1016/j.nmd.2017.05.015 28754454

[23] Teboul‐Coré S , Rey‐Jouvin C , Miquel A , et al. Bone imaging findings in genetic and acquired lipodystrophic syndromes: an imaging study of 24 cases. Skeletal Radiol. 2016;45:1495‐1506. 10.1007/s00256-016-2457-9 27631079

[24] Garg A . Gender differences in the prevalence of metabolic complications in familial partial lipodystrophy (Dunnigan variety). J Clin Endocrinol Metab. 2000;85:1776‐17782. 10.1210/jc.85.5.1776 10843151

[25] Patni N , Li X , Adams‐Huet B , Vasandani C , Gomez‐Diaz RA , Garg A . Regional body fat changes and metabolic complications in children with dunnigan lipodystrophy‐causing LMNA variants. J Clin Endocrinol Metab. 2019;104:1099‐1108. 10.1210/jc.2018-01922 30418556 PMC6382455

[26] Diker‐Cohen T , Cochran E , Gorden P , Brown RJ . Partial and generalized lipodystrophy: comparison of baseline characteristics and response to metreleptin. J Clin Endocrinol Metab. 2015;100(5):1802‐1810. 10.1210/jc.2014-4491 25734254 PMC4422900

[27] Oral EA , Gorden P , Cochran E , et al. Long‐term effectiveness and safety of metreleptin in the treatment of patients with partial lipodystrophy. Endocrine. 2019;64:500‐511. 10.1007/s12020-019-01862-8 30805888 PMC7340120

[28] Park JY , Javor ED , Cochran EK , DePaoli AM , Gorden P . Long‐term efficacy of leptin replacement in patients with Dunnigan‐type familial partial lipodystrophy. Metabolism. 2007;56(4):508‐516. 10.1016/j.metabol.2006.11.010 17379009 PMC2595136

[29] Simha V , Subramanyam L , Szczepaniak L , et al. Comparison of efficacy and safety of leptin replacement therapy in moderately and severely hypoleptinemic patients with familial partial lipodystrophy of the Dunnigan variety. J Clin Endocrinol Metab. 2012;97(3):785‐792. 10.1210/jc.2011-2229 22170723 PMC3319219

[30] Vatier C , Fetita S , Boudou P , et al. One‐year metreleptin improves insulin secretion in patients with diabetes linked to genetic lipodystrophic syndromes. Diabetes Obes Metab. 2016;18(7):693‐697. 10.1111/dom.12606 26584826

[31] Stears A , Hames C . Diagnosis and management of lipodystrophy: a practical update. Clin Lipidol. 2014;9(2):235‐259. 10.2217/clp.14.13

[32] Cole TJ . The LMS method for constructing normalized growth standards. Eur J Clin Nutr. 1990;44(1):45‐60. 2354692

[33] Haque WA , Shimomura I , Matsuzawa Y , Garg A . Serum adiponectin and leptin levels in patients with lipodystrophies. J Clin Endocrinol Metab. 2002;87:2395. 10.1210/jcem.87.5.8624 11994394

[34] Kothari S , Dhami‐Shah H , Shah SR . Antidiabetic drugs and statins in nonalcoholic fatty liver disease. J Clin Exp Hepatol. 2019;9(6):723‐730. 10.1016/j.jceh.2019.06.003 31889754 PMC6926203

[35] Crasto S , My I , Di Pasquale E . The broad spectrum of LMNA cardiac diseases: from molecular mechanisms to clinical phenotype. Front Physiol. 2020;11:761. 10.3389/fphys.2020.00761 32719615 PMC7349320

[36] Worman HJ . Nuclear lamins and laminopathies. J Pathol. 2012;226(2):316‐325. 10.1002/path.2999 21953297 PMC6673656

[37] Bonne G , Mercuri E , Muchir A , et al. Clinical and molecular genetic spectrum of autosomal dominant Emery‐Dreifuss muscular dystrophy due to mutations of the lamin A/C gene. Ann Neurol. 2000;48(2):170‐180. 10939567

[38] Fatkin D , MacRae C , Sasaki T , et al. Missense mutations in the rod domain of the lamin A/C gene as causes of dilated cardiomyopathy and conduction‐system disease. N Engl J Med. 1999;341(23):1715‐1724. 10.1056/NEJM199912023412302 10580070

